# Assessing the Performance of Land Consolidation Projects in Different Modes: A Case Study in Jianghan Plain of Hubei Province, China

**DOI:** 10.3390/ijerph17041410

**Published:** 2020-02-21

**Authors:** Bin Yang, Zhanqi Wang, Xiaowei Yao, Ji Chai

**Affiliations:** Department of Land Resource Management, School of Public Administration, China University of Geosciences (Wuhan), 388 Lumo Road, Hongshan District, Wuhan 430074, China; cugyangbin2011@163.com (B.Y.); xiaoweiyao@cug.edu.cn (X.Y.); chaiji_cug@163.com (J.C.)

**Keywords:** land consolidation, performance assessment, different modes, improved extensible matter–element model, Jianghan Plain

## Abstract

Land consolidation is essential in China for improving land use efficiency and supporting rural public space governance. Previously, the implementation of land consolidation projects (LCPs) had been mainly led by governments in China. In recent years, the Chinese government vigorously promoted land consolidation, and land consolidation in the corporation-leading mode (CLM) has emerged. This study focused on investigating the performance of CLM projects and the difference in performance levels between the CLM projects and the government-leading mode (GLM) projects. Based on the improved extensible matter–element model, the performance levels of 14 LCPs in GLM and CLM of Jianghan Plain, Hubei Province and related impact factors were analyzed. A set of evaluation indices was selected based on the “process-based thinking and logic”. Results showed that: (1) performance levels of the 14 LCPs are different, most of the projects in the GLM have ordinary or poor performance, while most of the projects in the CLM have excellent or good performance; (2) factors affecting the performance levels of LCPs are also different in the two modes. The main influencing factors in the CLM were the poor access to field roads, insufficient shelterbelt planting, and low land reclamation efficiency, while the insufficient shelterbelt planting, low annual output value of farmland and grain production capacity, low increase rate of agricultural labor production, and low land reclamation efficiency were the main influencing factors in the GLM; (3) comparative analysis of the two modes revealed that LCPs in the CLM have clear investment directions, high output benefits, and obvious advantages in the development of modern agriculture when compared with the GLM. To achieve improving the performance levels of LCPs, policy makers should actively innovate the implementation mode of LCPs and encourage all kinds of agricultural corporations to participate in land consolidation.

## 1. Introduction

Land consolidation began in the 14th century and the legislation appeared in the middle of the 18th century [1,2,3,4]. It is a tool for solving land fragmentation and increasing land use efficiency [5,6,7]. Along with land consolidation processes, it is also a useful tool for addressing the social, economic, public space governance, and sustainable development in rural area [8,9,10].

Land consolidation has been implemented in China since the mid-1990s with the purpose of increasing available cultivated land, reducing fragmentation, and promoting agricultural production capacity [11,12,13,14,15]. After more than 20 years of development, China’s land consolidation has entered the stage of comprehensive consolidation with mountains, rivers, forests, fields, lakes, and grasses [16,17]. It not only effectively alleviate the pressure of cultivated land protection and food security caused by the rapid industrialization and urbanization in China [18,19], but also improve the land productivity, farmers’ living conditions, the agricultural production, and rural development [20,21,22]. Land consolidation, with its unprecedented project scale, promotion scope, and investment intensity, has become an important issue and the focus of social attention in the field of public management in China [23,24]. It will continue to deeply affect the economic, social, and cultural development of rural areas in the future [25,26].

Land consolidation has been developing rapidly in China in the recent two decades. However, due to the fact that it was implemented as an experimental project, people focused on pursuing the quantity of cultivated land as the target, and the “top-down” planning and management mode was used, land consolidation in China generally has serious problems such as out-of-control and insufficient performance [27,28]. The evaluation of land consolidation projects (LCPs), therefore, is becoming a vital topic. Many researchers began to pay attention to the evaluation of LCPs and tried to design concepts and methods to conduct the evaluation of LCPs, including potential evaluation [29,30,31], comprehensive benefits evaluation [13,32,33], risks evaluation of society, ecology, and environment [34,35,36], and sustainable development for rural areas [37,38,39]. In recent years, some scholars tried to build an index system to evaluate the performance of LCPs from the perspective of farmers’ effective participation [40,41], farmers’ satisfactions and behaviors [42,43], project process and results [44,45], post-project management and maintenance [46,47]. Previous studies mainly focused on the performance levels measurement after the implementation of a single or a small number of LCPs, but rarely conducted comparative studies on the performance differences of LCPs. In addition, there were only a few quantitative studies on influencing factors of the performance from the perspective of single indicator level. As we know, evaluation results based on a single project usually have certain limitations and may contain contingency [48]. So, comparative analysis of multiple projects and multiple influencing factors may be of great value for improving LCPs performance by deeply exploring the performance differences and their influencing mechanisms.

In recent years, the Chinese government has vigorously promoted the innovation of land consolidation systems and mechanisms [24,49,50]. According to the national land consolidation plan (2016–2020) issued by the former ministry of land and resources and the national development and reform commission, farmers’ cooperatives, family farms, agricultural enterprises, and other new-type agricultural operation organizations are encouraged to participate in land consolidation in accordance with the principle of “government guidance, social participation, and policy guarantee”. In practice, some LCPs in the corporation-leading mode (CLM) have emerged. Previous studies paid more attention to the performance levels of LCPs in the traditional government-leading mode (GLM), while few studies have focused on performance levels of LCPs in CLM and the differences in performance levels of LCPs between GLM and CLM.

In this study, we attempted to investigate the performance levels of LCPs in both GLM and CLM and the differences between the two modes. The contributions of this study are divided into several sections. First, we built a performance evaluation index system of LCPs based on the method of “process thinking and process logic”. Second, we estimated the performance levels of 14 LCPs including GLM and CLM in Jianghan Plain of Hubei province by using the improved extensible matter–element model. Third, we analyzed the influencing factors of the 14 LCPs and explored the differences between the two modes. Lastly, we propounded some policy implications for improving the LCPs performance levels. The results of this work can not only enrich the method of LCPs performance assessment, but also can provide some scientific references for improving the performance levels of LCPs.

## 2. Study Area

Jianghan Plain is located in the middle reaches of the Yangtze River, central and southern Hubei Province, and covers Wuhan, Ezhou, Xiaogan, Xiantao, Qianjiang, Tianmen, Jingzhou, Jingmen, and other areas. The total area is 12,735.12 km^2^, accounting for 37.13 percent of the total area of Hubei Province. It had a resident population of 28.82 million and total GDP of 2156.36 billion Yuan in 2018, with the economic development level in the forefront of Hubei Province. Jianghan Plain is an important part of the Plain in the middle and lower reaches of the Yangtze River, with superior water and heat conditions, smooth terrain, fertile soil, and rich cultivated land resources. The total grain output in 2018 was 12.96 million tons, accounting for 47.95 percent of the total grain output in Hubei Province. It is also an important production base of grain, cotton, and oil in China.

**Figure 1:**
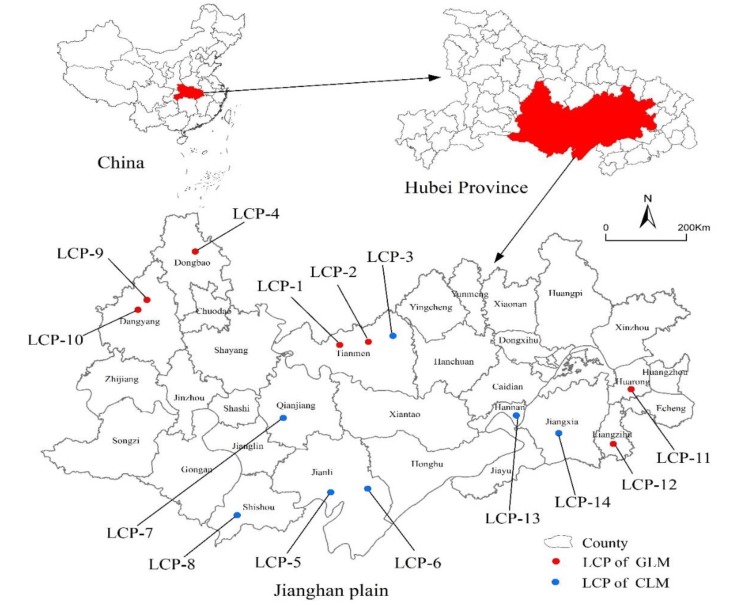
Locations of the study area and distributions of land consolidation projects (LCPs).

The selection of research objects should meet the following requirements: First, the topographical features, social economy, and agricultural production constraints of the region where the research objects are located must be consistent to ensure the comparability and accuracy of the data. Second, LCP types should include the GLM and CLM. Last, LCPs should be completed in recent 2–3 years so that the LCPs performance is not affected by the dilapidation and damage of projects in the later stage due to the long completion time. Based on the above principles, we chose 14 provincial-level investment projects that were completed in 2016–2017 and located in the hinterland of Jianghan Plain in Hubei Province as our study objects (Figure 1).

## 3. Methodology and Data Sources

### 3.1. Construction of a Performance Evaluation Index System

The implementation of LCP is a complex system engineering and its performance is not the same as the performance of traditional public projects [42]. Evaluation of its performance is based on the cumulative effect of a series of stages and processes of land consolidation and the comprehensive embodiment of the whole project implementation process and final results. Therefore, in this paper, we attempted to build a framework and index system for the performance evaluation of LCPs through the “process logic” theory. We searched for performance factors from the four aspects of a project including input, process, output, and effect. On the one hand, the investment and process dimensions represent the use of project funds, organization, and management rationality and efficiency in the implementation of a LCP, reflecting the process performance. On the other hand, the output and effect dimensions represent the actual output according to the project task and design and the effect brought by the project implementation, reflecting the result of a LCP. According to the four dimensions of “process logic” and the research results in earlier studies [48,51], we selected the project investment, project management, project construction, and project efficiency as the evaluation system layers and then selected the corresponding indicators. At last, the performance evaluation index system, including 13 individual indexes, was built (Table 1).

### 3.2. Performance Assessment Model for Land Consolidation Projects (LCPs)

There are many existing methods of LCPs performance assessments, such as multi-factor weighted assessment, principal components analysis, the ideal point method (TOPSIS), and so on. These methods have the advantage of easy operation by the ways of inputting all indicators simultaneously to calculate the results. However, they are often implanted with human subjective consciousness, and cannot reflect the “process thinking” of LCPs. So, there are some certain limitations of these methods. As a LCP is a complex system engineering, the implementation process usually needs to go through multiple links and processes, leading to its evaluation index has the characteristics of multi-dimensional and incompatibility [48]. For addressing the above problem, we tried to introduce the extensible matter–element model, which can not only be used for comprehensive evaluation to obtain the evaluation results, but also can analyze the performance affecting factors [44,52]. Moreover, it can reflect the process control characteristics to some extent and make sure that the evaluation results are more real and objective.

#### 3.2.1. Determining the Matter Element of LCPs Performance

Performance M of LCPs, performance characteristic c, and characteristic quantity value v jointly construct the performance matter element of LCPs. It is assumed that the performance M of LCPs is included n characteristics, which can be described by the characteristics $c_{1},\;c_{2},\ldots ,c_{n}$ and the corresponding magnitude values $v_{1},\;v_{2},\ldots ,v_{n}$. They can be expressed as:

(1) Rn=[Mc1v1 c2v2 ⋮⋮ cnvn]

In Equation (1), $R_{n}$ represents the n-dimensional performance of LCP, which can be abbreviated as $R_{n}=\left(M,c,v\right)$.

#### 3.2.2. Determining the Classic Domain of LCP Performance

The classic domain of LCPs performance can be expressed as:

(2) $$R_{0j}=\left(M_{0j},c,V_{0ji}\right)=\left[\begin{array}{ccc} M_{0j} & c_{1} & \langle a_{0j1},b_{0j1}\rangle \\ & c_{2} & \langle a_{0j2},b_{0j2}\rangle \\ & \vdots & \vdots \\ & c_{n} & \langle a_{0jn},b_{0jn}\rangle \end{array}\right]$$

In Equation (2), $R_{0j}$ is called the classical domain matter–element. $M_{0j}$ represents the evaluation level of j for the LCP performance that be divided; $c_{i}$ represents the evaluation index of i; interval $\langle a_{0ji},b_{0ji}\rangle$ represents the value range for the corresponding evaluation level of j for $c_{i}$; $V_{0ji}$ represents the value range $\langle a_{0ji},b_{0ji}\rangle$, that is the classic domain.

The determination of classical domain is the basis of matter–element evaluation for LCPs performance. According to the extensibility of matter–element evaluation, we divided the LCPs performance into four grades, namely, $T_{1}–T_{4}$ which were described as excellent, good, general, and poor, respectively. Taking the index data of many LCPs in the study area as a reference, and consulting opinions of related experts, the classic evaluation domain was finally determined, as shown in Table 2.

#### 3.2.3. Determining the General Domain of LCP Performance

The general domain of LCP performance can be expressed as:

(3) $$R_{p}=\left(P,C_{i},V_{ip}\right)=\left[\begin{array}{ccc} P & c_{1} & \langle a_{1p},b_{1p}\rangle \\ & c_{2} & \langle a_{2p},b_{2p}\rangle \\ & \vdots & \vdots \\ & c_{n} & \langle a_{np},b_{np}\rangle \end{array}\right]$$

In Equation (3), $R_{p}$ is called general domain matter–element; $V_{ip}=\langle a_{ip},b_{ip}\rangle \;\left(i=1,2\ldots ,n\right)$ represents value range of $c_{i}$ for general domain matter–element; P represents the whole evaluation grade of LCPs performance.

#### 3.2.4. Calculating the Correlation Function of Matter Element to Be Evaluated with Respect to Each Evaluation Grade

The correlation function of the classical matter–element model is segmented, so it is inconvenient to calculate the correlation degree. In addition, if the common domain or evaluation class of the characteristic indicators are different, their correlation degree cannot be compared. When the measured value exceeds the range of the common domain, the denominator of the correlation function in classical matter–element model is zero, which indicates the correlation degree cannot be calculated. Therefore, in order to solve the above problems, we combined the piecewise correlation functions of the classical matter–element model and defined a new hierarchical correlation function as follows:

(4) $$k_{ij}\left(x_{i}\right)=\frac{\frac{1}{2}\left(b_{ij}-a_{ij}\right)-\left|x_{i}-\frac{1}{2}\left(a_{ij}+b_{ij}\right)\right|}{\left(b_{ij}-a_{ij}\right)\left(b_{ip}-a_{i1}\right)}$$

In Equation (4), $a_{ij}$, $b_{ij}$ are the upper and lower limits of evaluation grade j on the classical domain as to the evaluation index i; $k_{ij}$ is the correlation degree of grade j with respect to $x_{i}$, j=1,2,3,4.

#### 3.2.5. Determining Weights of Evaluation Indicators

The weights of evaluation indicators are an important part for the matter–element evaluation. The rationality of weights is directly related to the accuracy and credibility of evaluation results. At present, the commonly used weight methods include the Delphi method, principal component analysis method, analytic hierarchy process (AHP), and entropy evaluation method. In this paper, the entropy evaluation method is used to determine the weights of evaluation indicators. The specific calculation results are shown in Table 2.

#### 3.2.6. Calculating the Comprehensive Relevance of the Matter–Element to Be Evaluated

The larger the correlation function value is, the closer the measured value is to the middle value of the evaluation grade interval. The value of the correlation function is positive, indicating that it is within the level interval. If it is negative, it means that it is not within the level interval, but there is still a chance to belong to the interval, and the larger the function value is, the more likely it is to be converted into the interval; the value of zero indicates a critical state in the level interval. After reasonably assigning weight to each evaluation indicator, the correlation degree of a research object to be evaluated $W_{x}$ respect to grade j can be expressed as:

(5) $$K_{j}\left(W_{x}\right)=\overset{m}{\underset{i=1}{\sum }}w_{i}k_{ij}\left(x_{i}\right)$$

In Equation (5), $K_{j}\left(W_{x}\right)$ represents the comprehensive correlation degree of $W_{x}$ about grade j, namely, the value of comprehensive performance. $k_{ij}\left(x_{i}\right)$ represents the correlation degree of evaluation index $x_{i}$ about grade j, namely, the value of single index performance. $w_{i}$ is the weight of the characteristic index of i.

### 3.3. Data Sources

The data of this paper mainly comes from the materials of LCPs, including documents of the project financial accounts and audits, documents of the summary of LCP supervision, documents of the expected benefits analysis of LCP investments, documents of the completion and acceptance summary of LCP, and so on. The specific data sources are shown in Table 3.

## 4. Results

### 4.1. Evaluation Results of Individual LCP Performance 

In order to analyze the performance level of a single project and its influencing factors, we took LCP-1 as an example. The index data of LCP-1 were substituted into the improved matter–element model, and the correlation degree of each index and the integral LCP-1 at each level could be obtained. According to the principle of maximum membership, the performance level of each indicator and the integral LCP-1 was judged, as shown in Table 4.

The evaluation results showed that the overall performance level of LCP-1 was general. From the perspective of single index, three of the 13 indexes achieved excellent performance levels, namely, investment per area $x_{1}$, budget execution deviation $x_{2}$, and the annual increased output value of farmland $x_{10}$. The performance levels of the four indexes were good, namely, deviation from schedule completion $x_{3}$, planning and design implementation $x_{4}$, accessibility of roads in the field $x_{7}$, and increased grain production capacity $x_{11}$. The performance levels of the five indexes were general, namely, increase rate of cultivated land area $x_{5}$, irrigation area increase rate $x_{6}$, density of protective forest network $x_{8}$, increased rate of land use $x_{9}$, increment of land reclamation coefficient $x_{13}$. In addition, there was one index with poor performance, which was the increased rate in agricultural labor production $x_{12}$.The reason why the performance level of this project was not excellent is that there were six individual indexes whose performance were general or below, which seriously hindered the overall performance level improvement of this project. Therefore, it can be generally concluded that the main influencing factors of LCP-1 performance level were low increase rate of cultivated land and irrigation area, insufficient shelterbelt planting, low land use improvement rate, and poor land reclamation coefficient.

### 4.2. Comparative Analysis of Performance Evaluation Results of LCPs in Different Modes

According to the improved matter–element evaluation model, we calculated the overall correlation degree of the other 13 LCPs and obtained their performance levels. The results were shown in Table 5.

Judging from the evaluation results, the performance levels of 14 LCPs in Jianghan Plain were different, with large differences among them. Performance levels of most LCPs in the GLM were general or poor, while performance levels of most LCPs in the CLM were excellent or good. Specifically, in the GLM, one project had good performance level, which was LCP-12; two projects were at general performance levels, which were LCP-1 and LCP-9, respectively; four projects were at poor performance levels, which were LCP-2, LCP-4, LCP-10, and LCP-11, respectively. While in the CLM, three projects had excellent performance levels, which were LCP-3, LCP-6, and LCP-8, respectively; three projects were at good performance levels, which were LCP-5, LCP-7, and LCP-13, respectively; one project had general performance level, which was LCP-14. It can be seen that LCPs performance levels varied greatly in the two modes, and performance level in the CLM was higher than that in the GLM on the whole.

### 4.3. Analysis of Influencing Factors

Compared with other evaluation methods, matter–element evaluation model not only can reveal the integral performance level of one project, but also can evaluate performance level of a single index. The calculation and evaluation of single index performance level can be used to quantitatively analyze the influencing factors of LCPs performance and reflect the problems existing in LCPs. According to the evaluation results of single index, the proportion of evaluation objects in the four grades of each index in the two modes was statistically analyzed, and the main influencing factors of LCPs performance, namely obstacle degree, can be obtained. It is stipulated that if an evaluation index takes up more than half of the obstruction rating (general and poor degrees are defined as obstruction rating), it indicates that this index is the obstruction factor for most projects. The statistical results were shown in Table 6.

It can be seen from Table 6 that the obstacle degree of road network density, shelter forest density and land reclamation coefficient increment in the CLM reached 71.43%, 57.14%, and 100.00%, respectively. Therefore, it can be generally considered that the poor accessibility of field roads, insufficient shelterbelt planting, and low land reclamation coefficient were the main influencing factors of LCPs performance level in the CLM. In the GLM, the obstacle degrees of shelter forest density, increase of annual output value of farmland, increase of grain production capacity, increase rate of agricultural labor production, and increase rate of land reclamation coefficient reached 71.43%, 57.14%, 57.14%, 71.43%, and 100.00%, respectively. So, it can be generally considered that insufficient shelterbelt planting, low annual output value of farmland and grain production capacity, low increase rate of agricultural labor production, and low land reclamation coefficient were the main influencing factors of LCPs performance level in the GLM. As a whole, there were many significant differences in the influencing factors of LCPs performance level between the two modes.

Land consolidation in China has basically adopted the GLM since its implement in the mid-1990s. In this mode, the governments are not only the investors, but also the main organizers and implementers of the projects. They only need to complete the tasks, and the phenomenon of “coping tasks” is common in the GLM. In the meantime, the main beneficiary and investment implementation party are not consistent, which causes insufficient incentive for investors and low investment efficiency. Due to lack of land transfer in this mode, field roads, irrigation and water conservancy facilities, and other engineering facilities can only meet the agricultural production needs of a single family which leads to the degree of integration with large-scale industrial development not being enough, as existing problems such as consolidation mode tend to assimilate, the overall positioning is low, and the coordination is limited [49]. In addition, farmers’ participation to LCP is not enough, which is not conducive to promote the local farmers’ employment and the agricultural labor productivity is relatively low. These factors hinder the improvement of performance levels of LCPs.

On the contrary, in the CLM, the investment from social organizations is not restrained by time and space. In this case, the organization, management, and implementation of LCPs are all undertaken by the agricultural corporations. Local land management departments can better perform their functions of instruction and supervision. Moreover, the tendering and bidding steps are canceled in this mode and agricultural corporations arrange the engineering schedule on their own. This can not only greatly shorten the construction preparation period and win favorable golden construction period for LCPs, but also save the upfront work costs and project supervision costs which can input to the project construction. At the same time, the work of land flatness in project construction is relatively high, and the quality of irrigation and water conservancy facilities are relatively good. These may be conducive to the development of modern agriculture and can improve the agricultural labor productivity in the project construction area [53,54]. The above factors can greatly contribute to improving the performance levels of LCPs.

## 5. Discussion

### 5.1. The Differences between the Two Modes of LCPs

The government-leading mode (GLM) is a “top-down” implementation mode based on administrative management. Local governments are the owners of LCPs. They are not only the investors but also the implementers and main supervisors of LCPs. The procedures of LCPs in GLM are as follows: Firstly, the survey and investigation teams composed of the staff and relevant experts from the land, agriculture, and other departments in the related local government conduct an investigation and demonstration of the project area and determine the construction projects. Then, the local government invites project design companies to bid on the project, and the winning design company then carries out the feasibility study, project planning, and design and budget compilation as required. After this, the local government bides the project for construction and supervision work, and then the winning construction company carries out the project construction while the winning supervision company conducts the construction supervision. Lastly, the local county government organizes a preliminary inspection of the project, and the provincial department of land management organizes some experts to conduct the final approval inspection after the project completion. This mode is formulated by the “upper layer”, while the “lower layer” of land users and farmers who are the ultimate beneficiaries of LCPs are in a passive position. Therefore, it is difficult to fully reflect the production needs of land users and farmers. All the time, LCPs have basically adopted this “top-down” GLM in China.

The corporation-leading mode (CLM), which is based on the leading role of newly emerged agricultural corporations in land consolidation, is a “bottom-up” implementation mode of LCPs. In this mode, a LCP is carried out by local agricultural corporations or farmers’ professional cooperatives, which are organizers and implementers of the project, while the government only provides corresponding technical guidance and supervision. The implementation procedures of LCPs in the CLM are as follows: Firstly, agricultural corporations or farmers’ professional cooperatives submit a written application for a LCP to the local government. The local government organizes some relevant experts to survey the site and conducts a feasibility study to determine whether the project could be carried out or not. Then, agricultural corporations or farmers’ professional cooperatives formulate the project implementation plan (including planning and design, budget, construction plan, etc.) and submit it to the local government for examination and approval. Agricultural corporations or farmers’ professional cooperatives implement the LCP independently in accordance with the approved implementation plan. Lastly, the local government organizes some experts to conduct the final acceptance inspection and gives some certain subsidies to them. The implementation of this mode is based on the premise that agricultural corporations or farmers’ professional cooperatives sign contracts with the local farmers for land transfer. It will help to realize the integration of land consolidation and agricultural industry projects. According to the “National Land Consolidation Plan (2016–2020)”, farmers’ cooperatives, family farms, agricultural corporations, and other new business entities are encouraged to participate in LCPs in accordance with the principle of “government guidance, society participation, and policy guarantee”. In recent years, some local governments have carried out the pilot LCPs in the CLM. Nevertheless, LCPs implemented in this mode are still relatively few in China.

### 5.2. Policy Implications for Improving the Performance Levels of LCPs

As analyzed above, the CLM is more flexible and has more advantages than GLM. To achieve improving the performance levels of LCPs, policy makers should actively innovate the implementation mode of LCP and encourage all kinds of agricultural corporations to participate in land consolidation. Some policy implications are as follows:

- (i) The principle of “construction first and supplement after, and promoting construction by supplement” can be adopted for land consolidation in the CLM. Agricultural corporations invest in LCP first, and the government provides some subsidies according to certain standards after LCP is inspected as qualified. In this way, it can better strengthen project construction and guarantee that the investment to agricultural corporations in total is no less than a certain proportion.
- (ii) We should continue to deepen the reform of the rural land system, improve the “three rights separation” system for rural land and conduct transfer of agricultural land by following the principle of farmers’ own voluntaries. In addition, the cultivation of new types of agricultural operators such as family farms and farmers’ cooperatives should be given priority to lay some foundations for launching comprehensive land consolidation and developing modern agriculture.
- (iii) The agricultural corporations can be encouraged to take the increased cultivated land by reclamation of the waste rural residential areas and consolidation of fragmented cultivated land as the indicators of occupying and replenishing cultivated land in a balanced way. According to the principle of “who invests, who benefits”, the indicator trading incomes are returned to broaden the fund source channels for land consolidation in the CLM.
- (iv) The local government should increase the technical guidance and supervisions in the whole process of the CLM. By this way, on the one hand, it can provide support and guarantee for the legal implementation of LCPs by agricultural corporations, on the other hand, it can prevent agricultural corporations from illegally obtaining funds from LCPs.

### 5.3. Study Limitations and Further Research Prospects

In this paper, we built a LCP performance evaluation index system based on the method of “process thinking and process logic” and introduced the improved extensible matter–element model to assess the performance levels and analyze the influence factors of 14 LCPs including the GLM and CLM in Jianghan Plain, Hubei province. Our study may have some important implications for improving the method of LCP performance assessment and be helpful to strengthen the administration of public health in rural areas through the implementation of LCPs. However, there are inevitably some problems that need to be solved in further research. Due to the limitation of data collection, we only selected 14 projects in the plain type area of Hubei Province as research objects to demonstrate the evaluation index system and the improved extensible matter–element model, whose rationality has yet to be verified by other topographic areas, such as hills and mountains. Moreover, LCP is a complex system engineering, and its performance connotation is much richer than the measurement index selected in this study. In further research, it is necessary to analyze the performance connotation of LCP more deeply and construct a richer index system to comprehensively evaluate LCP performance in different modes. Last but not least, as LCPs can effectively promote the management of public space in rural areas and the construction of a green and healthy habitat environment through the methods of production space management, urban, and rural overall development, ecological environment governance, and extensive participation of farmers [55,56], so it may be another important direction for future research to explore the impact mechanism and its coupling relationship on LCP and public space governance to ensure the healthy and stable development of rural areas.

## 6. Conclusions

In this study, a LCP performance evaluation index system was established based on the method of “process thinking and process logic”. Meanwhile, the improved extensible matter–element model was introduced to assess the performance levels and analyze the influence factors of 14 LCPs implemented with the GLM and CLM in Jianghan Plain, Hubei province. The conclusions of this study are summarized as follows.

The performance levels of 14 LCPs were different from each other. Most of the LCPs in the GLM had general or poor performance, while most of the LCPs in the CLM had good or excellent performance. Among them, one LCP had good performance level, two projects had general performance level and four projects had poor performance level in the GLM. In contrast, there were three LCPs with excellent performance level, three LCPs with good performance level, and one LCP with general performance level in the CLM.

Further analysis showed that the influencing factors of performance levels of the 14 LCPs were also different in the two modes. The main influencing factors in the CLM were poor access to field roads, insufficient shelterbelt planting, and low land reclamation efficiency, while the insufficient shelterbelt planting, low annual output value of farmland and grain production capacity, low increase rate of agricultural labor production, and low land reclamation efficiency were the main influencing factors in the GLM.

Comparative analysis of the two modes revealed that LCPs in the CLM have clear investment directions, high output benefits, and obvious advantages in the development of modern agriculture when compared with the GLM. To achieve improving the performance levels of LCPs, policy makers should actively innovate the implementation mode of LCPs and encourage all kinds of agricultural corporations to participate in land consolidation. Above all, this paper proposes that the performance evaluation index system and the improved extensible matter–element model should be used for the performance evaluation and influencing factors analysis of LCPs to provide new ideas for improvement of LCP performance and public project management, and more references for decision making.

## Figures and Tables

**Table 1:** The performance evaluation index system of land consolidation projects and its weight.

The Target Layer	Rule Layer	Index Layer	Definition of Indicator	Weight
Performance of LCPs	Investment	Investment per area $x_{1}$/($\mathrm{yuan}\cdot {\mathrm{hm}}^{-2}$)	$x_{1}=\frac{CI}{AI}$, where CI is the amount of land consolidation investment, AI is the area of LCP.	0.0221
	Management	Budget execution deviation $x_{2}$/%	$x_{2}=\frac{F1-F2}{F2}\times 100\%$, where F1 is the actual funding, F2 is the total budget funding.	0.0289
		Deviation from schedule completion $x_{3}$/%	$x_{3}=\frac{D1-D2}{D2}\times 100\%$, where D1 is the actual completion days, D2 is the planned completion days.	0.0212
		Planning and design Implementation $x_{4}$/%	$x_{4}$ is a score according to the opinions of some experts.	0.0510
	Construction	Increase rate of cultivated land area $x_{5}$/%	$x_{5}=\frac{S1-S2}{S2}\times 100\%$, where S1 is the cultivated land area after implementation of LCP, S2 is the cultivated land area before implementation of LCP.	0.1193
		Irrigation area increase rate $x_{6}$/%	$x_{6}=\frac{S1-S2}{S2}\times 100\%$, where S1 is the irrigation area after implementation of LCP, S2 is the irrigation area before implementation of LCP.	0.0698
		Accessibility of roads in the field $x_{7}/\left(m\cdot {\mathrm{hm}}^{-2}\right)$	$x_{7}$ = $\frac{LI}{AI}$, where $x_{7}$ represents the accessibility of roads in the field; LI is the total length of farmland roads and production roads in the project area; AI is the total area of LCP.	0.0296
		Density of protective forest network $x_{8}/\left(\mathrm{plant}\cdot {\mathrm{hm}}^{-2}\right)$	$x_{8}=\frac{NI}{AI}$, where NI is the total number of shelterbelts in the LCP, AI is the total area of LCP.	0.0979
	Efficiency	Increased rate of land use $x_{9}$/%	$x_{9}=\frac{S1-S2}{S2}\times 100\%$, where S1 is the available land area after implementation of LCP, S2 is the available land area before implementation of LCP.	0.1217
		The annual increased output value of farmland $x_{10}/\left(\mathrm{yuan}\cdot {\mathrm{hm}}^{-2}\right)$	$x_{10}=\frac{V1-V2}{AI}$, where V1 is the annual output value of farmland after implementation of LCP, V2 is the annual output value of farmland before implementation of LCP, AI is the total area of LCP.	0.1544
		Increased grain production capacity $x_{11}/\left(\mathrm{kg}\cdot {\mathrm{hm}}^{-2}\right)$	$x_{11}=\frac{G1-G2}{AI}$, G1 is the grain yield after implementation of LCP, G2 is the grain yield before implementation of LCP, AI is the total area of LCP.	0.1215
		Increased rate in agricultural labor production $x_{12}$/%	$x_{12}=\frac{P1-P2}{P2}\times 100\%$, P1 is the agricultural labor productivity after implementation of LCP, P2 is the agricultural labor productivity before implementation of LCP.	0.0887
		Increment of land reclamation coefficient $x_{13}$/%	$x_{13}=\frac{CI}{AI}\times 100\%$, CI is the increased cultivated land area, AI is the total area of LCP.	0.0739

**Table 2:** Scale in classic domain of performance evaluation indexes of LCPs.

Indicators and Their Units	Value Range			
	Excellent	Good	General	Poor
$x_{1}$: Investment per area/(yuan∙hm^−2^)	[10,000,20,000)	[20,000,25,000)	[25,000,30,000)	[30,000,35,000)
$x_{2}$: Budget execution deviation/%	[0,5)	[5,20)	[20,50)	[50,100)
$x_{3}$: Deviation from schedule completion/%	[0,10)	[10,20)	[20,50)	[50,100)
$x_{4}$: Planning and design Implementation/score	[90,100)	[75,90)	[60,75)	[50,60)
$x_{5}$: Increase rate of cultivated land area/%	[5,10)	[2.5,5)	[1,2.5)	[0,1)
$x_{6}$: Irrigation area increase rate/%	[20,30)	[10,20)	[5,10)	[0,5)
$x_{7}$: Accessibility of roads in the field/(m∙hm^−2^)	[150,200)	[100,150)	[50,100)	[0,50)
$x_{8}$: Density of protective forest network/(plant∙hm^−2^)	[75,100)	[50,75)	[25,50)	[0,25)
$x_{9}$: Increased rate of land use/%	[10,15)	[5,10)	[2,5)	[0,2)
$x_{10}$: The annual increased output value of farmland/(yuan∙hm^−2^)	[4000,6000)	[2500,4000)	[1000,2500)	[0,1000)
$x_{11}$: Increased grain production capacity/(kg∙hm^−2^)	[2000,3000)	[1500,2000)	[1000,1500)	[0,1000)
$x_{12}$: Increased rate in agricultural labor production/%	[10,20)	[5,10)	[2,5)	[0,2)
$x_{13}$: Increment of land reclamation coefficient/%	[10,20)	[5,10)	[2,5)	[0,2)

**Table 3:** Data sources for the indicators in this paper.

Indicators	Data Sources	Data Format	Date
$x_{1}$	Documents of the project financial accounts and audits	PDF	2016.09
$x_{2}$	Documents of the project financial accounts and audits	PDF	2016.09
$x_{3}$	Documents of the summary of LCP supervision	PDF	2016.11
$x_{4}$	Given by some experts.	Score	2017.04
$x_{5}$	Documents of the expected benefits analysis of LCP investments	PDF	2016.12
$x_{6}$	Documents of the expected benefits analysis of LCP investments	PDF	2016.12
$x_{7}$	Documents of the expected benefits analysis of LCP investments	PDF	2016.12
$x_{8}$	Documents of the expected benefits analysis of LCP investments	PDF	2016.12
$x_{9}$	Documents of the completion and acceptance summary of LCP	PDF	2017.04
$x_{10}$	Documents of the completion and acceptance summary of LCP	PDF	2017.04
$x_{11}$	Documents of the completion and acceptance summary of LCP	PDF	2017.04
$x_{12}$	Documents of the completion and acceptance summary of LCP	PDF	2017.04
$x_{13}$	Documents of the completion and acceptance summary of LCP	PDF	2017.04

**Table 4:** Results of performance evaluation of LCP-1.

Evaluating Indicators or Object	Correlation Degree				Performance Level
	$K_{1}\left(x_{i}\right)/K_{1}\left(M_{x}\right)$	$K_{2}\left(x_{i}\right)/K_{2}\left(M_{x}\right)$	$K_{3}\left(x_{i}\right)/K_{3}\left(M_{x}\right)$	$K_{4}\left(x_{i}\right)/K_{4}\left(M_{x}\right)$	
$x_{1}$	0.00001	−0.00004	−0.00044	−0.00084	Excellent
$x_{2}$	0.00000	−0.00333	−0.00667	−0.01000	Excellent
$x_{3}$	−0.00400	0.00400	−0.00200	−0.00720	Good
$x_{4}$	−0.02000	0.01167	−0.01167	−0.05500	Good
$x_{5}$	−0.05587	−0.01174	0.01956	−0.12066	General
$x_{6}$	−0.04529	−0.01196	0.00942	−0.00942	General
$x_{7}$	−0.00414	0.00086	−0.00086	−0.00586	Good
$x_{8}$	−0.01612	−0.00612	0.00388	−0.00388	General
$x_{9}$	−0.09027	−0.02360	0.02733	−0.04100	General
$x_{10}$	−0.00005	−0.00029	−0.00046	−0.00094	Excellent
$x_{11}$	−0.00007	0.00013	−0.00020	−0.00027	Good
$x_{12}$	−0.04151	−0.03302	−0.00503	0.00755	Poor
$x_{13}$	−0.03885	−0.02770	0.00383	−0.00575	General
LCP-1	−0.03019	−0.01010	0.00558	−0.02378	General

Note: $K_{j}\left(c_{i}\right)\left(i=1,2,\cdots ,14\right)$ represents the correlation degree of indicator $c_{i}$ at grade j; $K_{j}\left(M_{1}\right)$ represents the comprehensive correlation degree of the evaluation object $M_{1}$ at grade j.

**Table 5:** Results of performance evaluation of 14 LCPs in Jianghan Plain of Hubei province.

Project Number	Correlation Degree				Performance Level
	$K_{1}\left(M_{x}\right)$	$K_{2}\left(M_{x}\right)$	$K_{3}\left(M_{x}\right)$	$K_{4}\left(M_{x}\right)$	
LCP1-GLM	−0.03019	−0.01010	0.00558	−0.02378	General
LCP2-GLM	−0.03952	−0.02237	−0.00921	0.00076	Poor
LCP3-CLM	0.00322	−0.01923	−0.07266	−0.14262	Excellent
LCP4-GLM	−0.03667	−0.01959	−0.00425	−0.00241	Poor
LCP5-CLM	−0.02333	−0.00681	−0.01027	−0.05355	Good
LCP6-CLM	−0.02665	−0.03900	−0.07904	−0.10967	Excellent
LCP7-CLM	−0.02247	−0.00530	−0.00627	−0.04078	Good
LCP8-CLM	−0.01415	−0.01872	−0.03681	−0.06815	Excellent
LCP9-GLM	−0.03160	−0.01412	0.00572	−0.01750	General
LCP10-GLM	−0.03777	−0.02553	−0.01114	−0.00184	Poor
LCP11-GLM	−0.04047	−0.01930	−0.00865	−0.00725	Poor
LCP12-GLM	−0.02143	−0.00416	−0.01447	−0.05922	Good
LCP13-CLM	−0.02207	−0.01219	−0.02529	−0.04369	Good
LCP14-CLM	−0.03699	−0.02169	−0.00559	−0.00769	General

**Table 6:** Statistic of influencing factors of performance of LCPs in the two modes.

Evaluating Indicators	The Proportion of GLM Projects at Different Levels/%				Obstacle Degree/%	The Proportion of CLM Projects at Different Levels/%				Obstacle Degree/%
	Excellent	Good	General	Poor		Excellent	Good	General	Poor	
$x_{1}$	42.86	14.29	28.57	14.29	42.86	14.29	71.43	0.00	14.29	14.29
$x_{2}$	57.14	14.29	28.57	0.0	28.57	42.86	28.57	28.57	0.00	28.57
$x_{3}$	28.57	28.57	28.57	14.29	42.86	42.86	42.86	14.29	0.00	14.29
$x_{4}$	28.57	57.14	14.29	0.00	14.29	71.43	28.57	0.00	0.00	0.00
$x_{5}$	42.86	14.29	28.57	14.29	42.86	71.43	0.00	28.57	0.00	28.57
$x_{6}$	28.57	28.57	28.57	14.29	42.86	28.57	57.14	0.00	14.29	14.29
$x_{7}$	57.14	14.29	14.29	14.29	28.57	0.00	28.57	42.86	28.57	71.43
$x_{8}$	14.29	14.29	28.57	42.86	71.43	14.29	28.57	42.86	14.29	57.14
$x_{9}$	14.29	42.86	28.57	14.29	42.86	42.86	28.57	28.57	0.00	28.57
$x_{10}$	42.86	0.00	28.57	28.57	57.14	57.14	42.86	0.00	0.00	0.00
$x_{11}$	14.29	28.57	28.57	28.57	57.14	42.86	57.14	0.00	0.00	0.00
$x_{12}$	14.29	14.29	14.29	57.14	71.43	71.43	14.29	14.29	0.00	14.29
$x_{13}$	0.00	0.00	28.57	71.43	100.00	0.00	0.00	42.86	57.14	100.00
